# Supplementary material for: Rosmanol and Carnosol Synergistically Alleviate Rheumatoid Arthritis through Inhibiting TLR4/NF-κB/MAPK Pathway
Source: Molecules. 2021 Dec 23;27(1):78. doi: 10.3390/molecules27010078 (PMC8746366; doi:10.3390/molecules27010078)
Supplement: Supplementary file 1 [file molecules-27-00078-s001.zip › molecules-1475710-supplementary.pdf]

## The contents determination of rosmanol and carnosol in *Callicarpa longissima*

**Chromatographic conditions**: The standard reference materials and methanol extract of *Callicarpa longissima* were analyzed by LC-2030C HPLC (SHIMADZU, Japan) with C18 column (250 × 4.6 mm, 5μm) at 30℃. The mobile phase, composed of acetonitrile (A) and 0.1% phosphoric acid (B), was applied in gradient elution model as follows: 0-35 min, 35%A-62%A. The flow rate was kept at 0.8 ml/min and detection wavelength was set at 210 nm.

**Preparation of standard reference solution**: Rosmanol (4.69 mg) and carnosol (4.04 mg) were dissolved and quantified in 50 ml volumetric flask with methanol.

**Preparation of sample solution**: 2g powder of branches and leaves of *Callicarpa longissima* was extract with methanol by ultrasonic extractor for 1 h, and the filtered extract solution was quantified in 50 ml volumetric flash with methanol.

**Contents determination**: The stand curves of rosmanol and carnosol were established and used to calculate for contents based on their peak areas in chromatogram of methanol extract of *Callicarpa longissima*. The results showed that contents of rosmanol and carnosol were 0.46 and 2.37 mg/g, respectively.

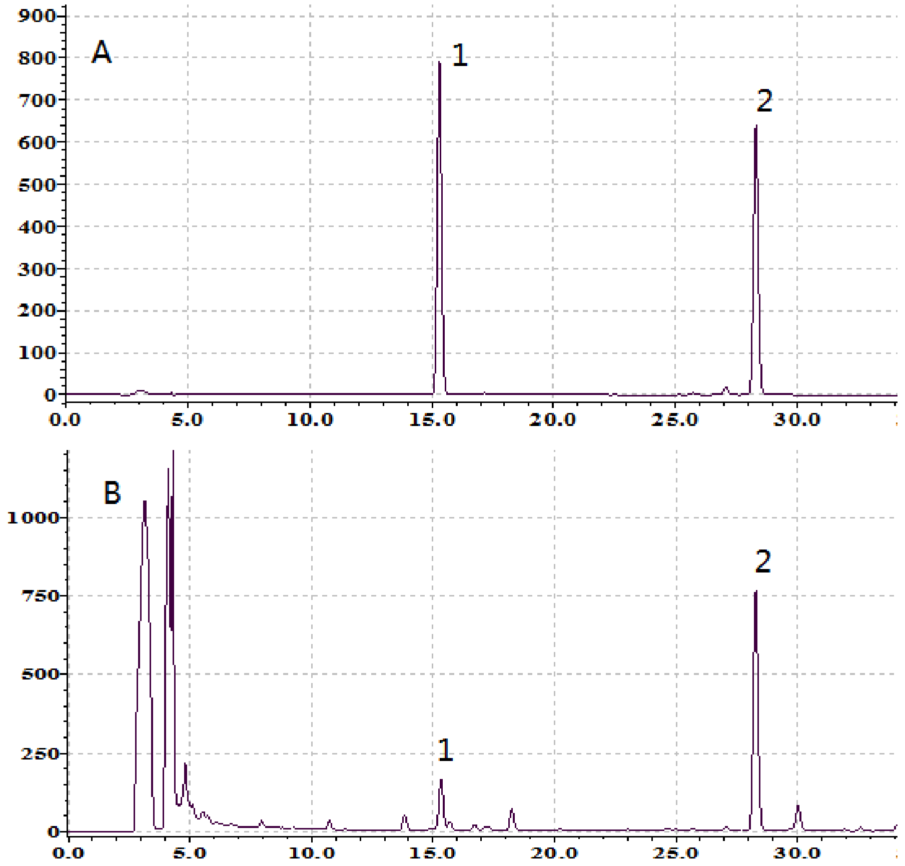


Figure S1. (A) Chromatogram of standard reference materials, in which peak 1, 2 were rosmanol and carnosol, respectively. (B) Chromatogram of methanol extract of *Callicarpa longissima*.

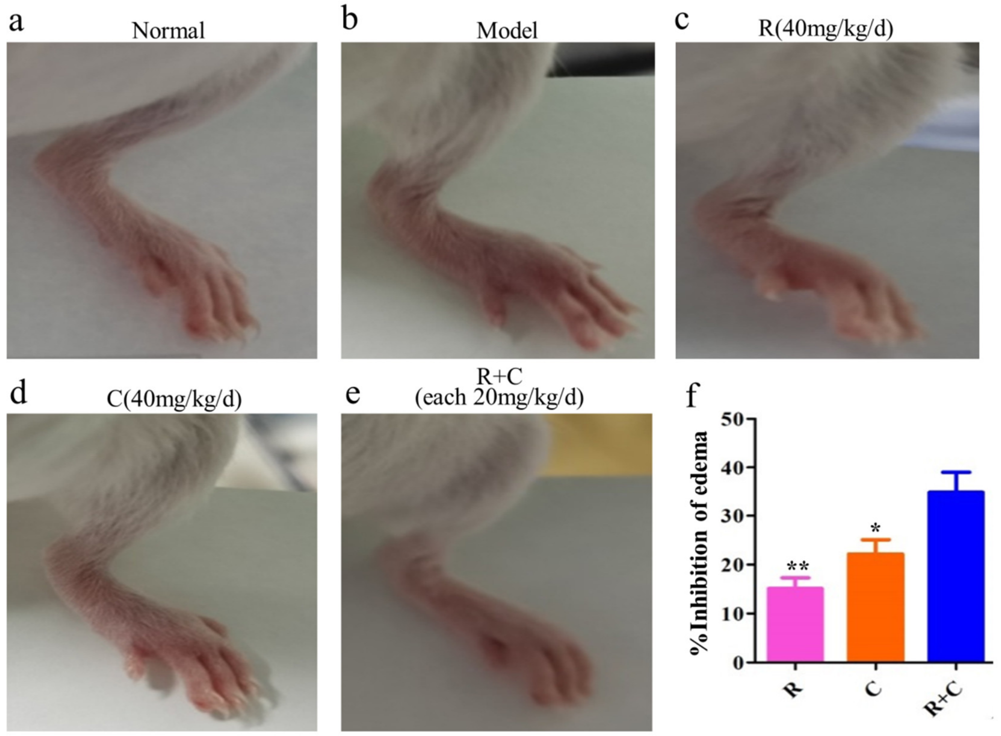


Figure S2. Rosmanol and carnosol synergistically inhibited paw edema in carrageenan-induced Kunming mice model. Paw edema was induced with 1% (w/v) carrageenan suspension by subcutaneous injection. Rosmanol (40 mg/kg), carnosol (40 mg/kg) and their combination (20 mg/kg rosmanol and 20 mg/kg carnosol) were administered by intragastric gavage 1 h before subcutaneous injection. Representative pictures from the left hind limb of the normal group (a), model group (b), rosmanol-treated group (c), carnosol-treated group (d) and combination-treated group (f). Inhibitory rate of rosmanol (R), carnosol (C) and their combination (R+C) on paw edema (f). Data were presented as the mean ± sd (n = 6). The significance of differences was analyzed by unpaired t-test. *, $p<0.05$ *vs* the combination-treated group; **, $p<0.01$ *vs* the combination-treated group.

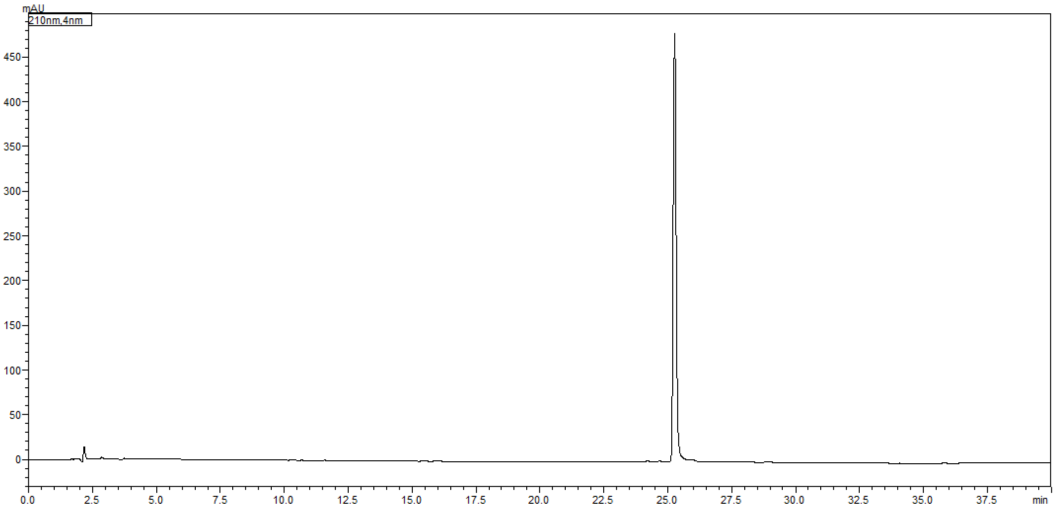


Figure S3. HPLC spectrum of rosmanol (**1**).

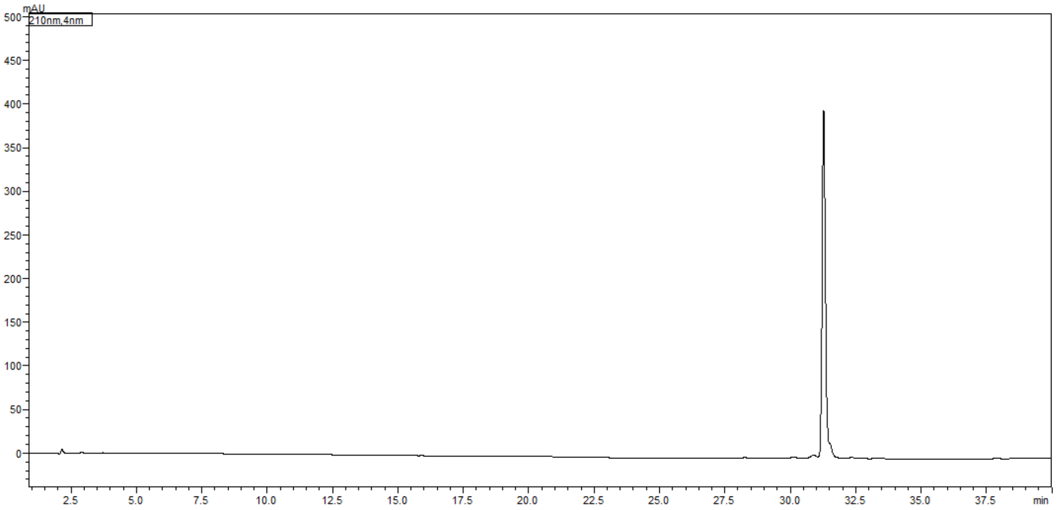


Figure S4. HPLC spectrum of carnosol (**2**).

## Structure elucidation

Compound **1,** obtained as white powder, had the molecular formula $C_{20}H_{26}O_5$ on basis of HR-ESI-MS at m/z 345.1688 $[M-H]^-$. $^1$H-NMR (500 MHz, $CD_3OD$), $\delta_H$: 6.84 (1H, s, H-14)，4.59 (1H, d, $J$ = 3.5 Hz, H-6α), 4.52 (1H, d, $J$ = 3.5 Hz, H-7β), 3.29 (1H, m, H-1β), 3.19 (1H, m, H-15), 2.26 (1H, s, H-5), 1.94 (1H, td, $J$ = 14.0, 5.5 Hz, H-1α), 1.60 (1H, m, H-2β), 1.44 (2H, m, H-2α, 3β), 1.23 (1H, td, 13.5, 3.0 Hz, H-3α), 1.21 (3H, d, $J$ = 7.0 Hz, H-16), 1.18 (3H, d, $J$ = 7.0 Hz, H-17), 1.03 (3H, s, H-18), 0.91 (3H, s, H-19); $^{13}$C-NMR (125 MHz, $CD_3OD$), $\delta_C$: 28.7 (C-1), 20.2 (C-2), 39.5 (C-3), 32.4 (C-4), 51.7 (C-5), 80.0 (C-6), 69.3 (C-7), 129.5 (C-8), 125.1 (C-9), 48.4 (C-10), 145.4 (C-11), 143.5 (C-12), 137.5 (C-13), 120.5 (C-14), 28.0 (C-15), 23.0 (C-16), 23.2 (C-17), 31.9 (C-18), 22.5 (C-19), 181.0 (C-20). The NMR spectral data of **1** were in good agreement with those of rosmanol reported in the reference, so was identified as rosmanol.

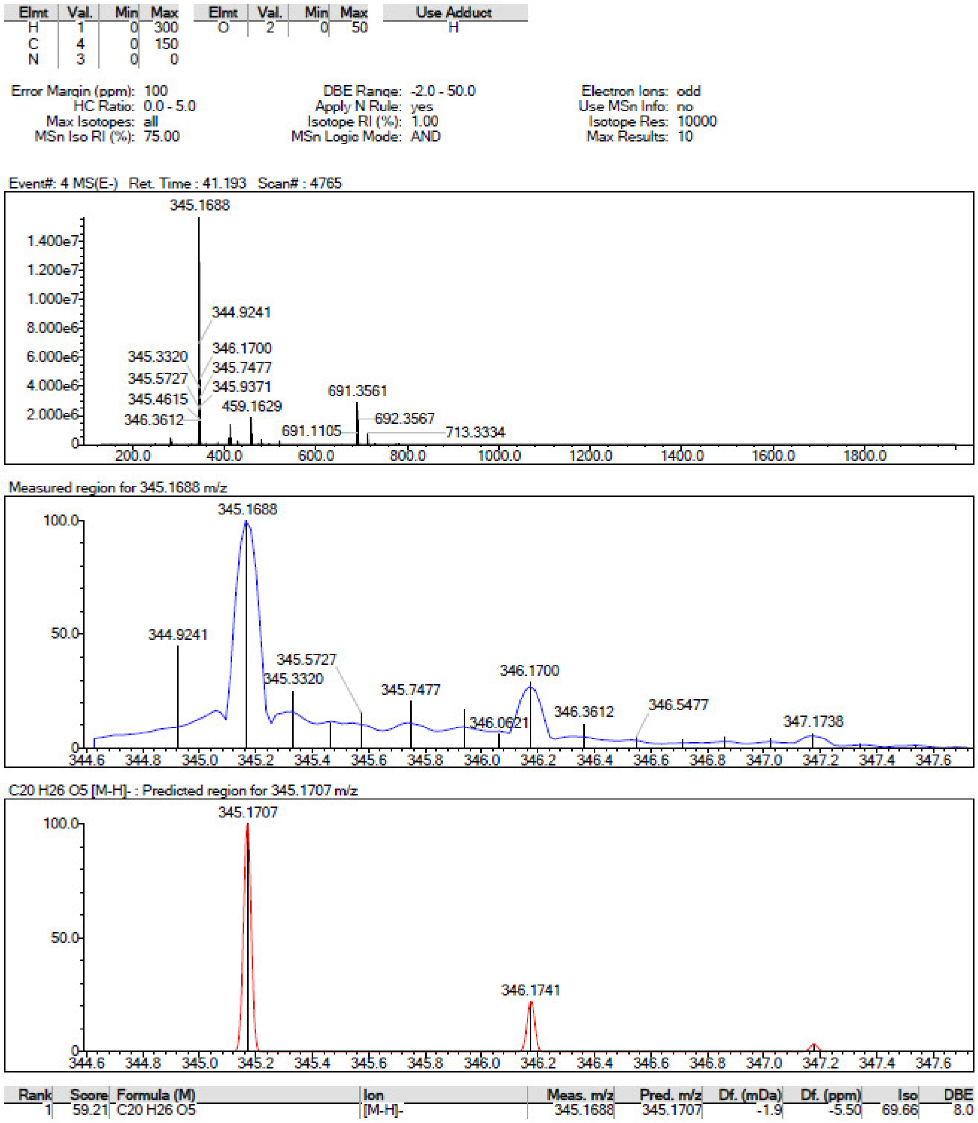


Figure S5. HR-ESI-MS spectrum of rosmanol (**1**).

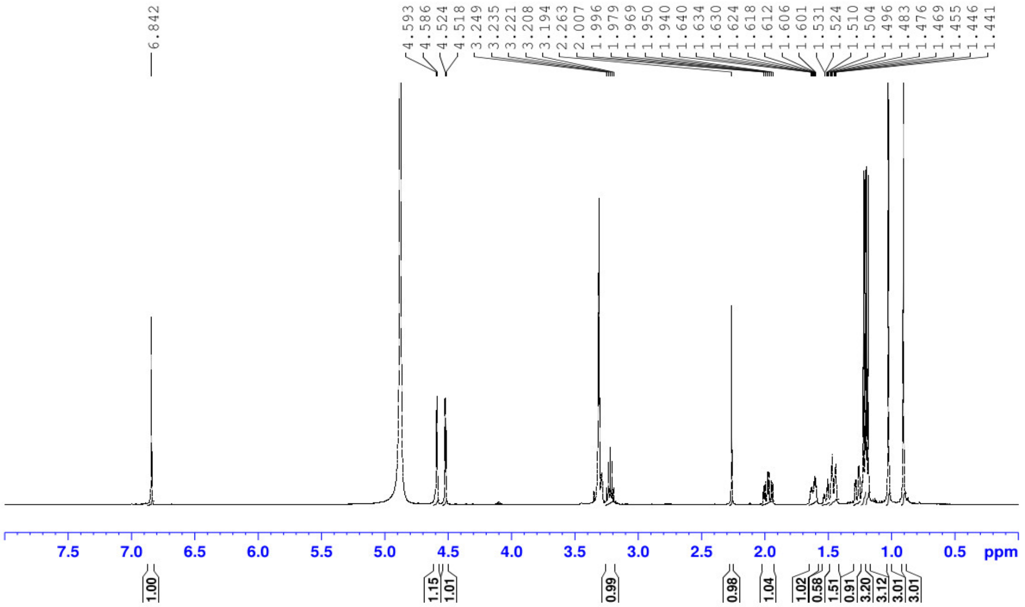


Figure S6. $^{1}$H-NMR spectrum of rosmanol (**1**) (500 MHz in $CD_3OD$).

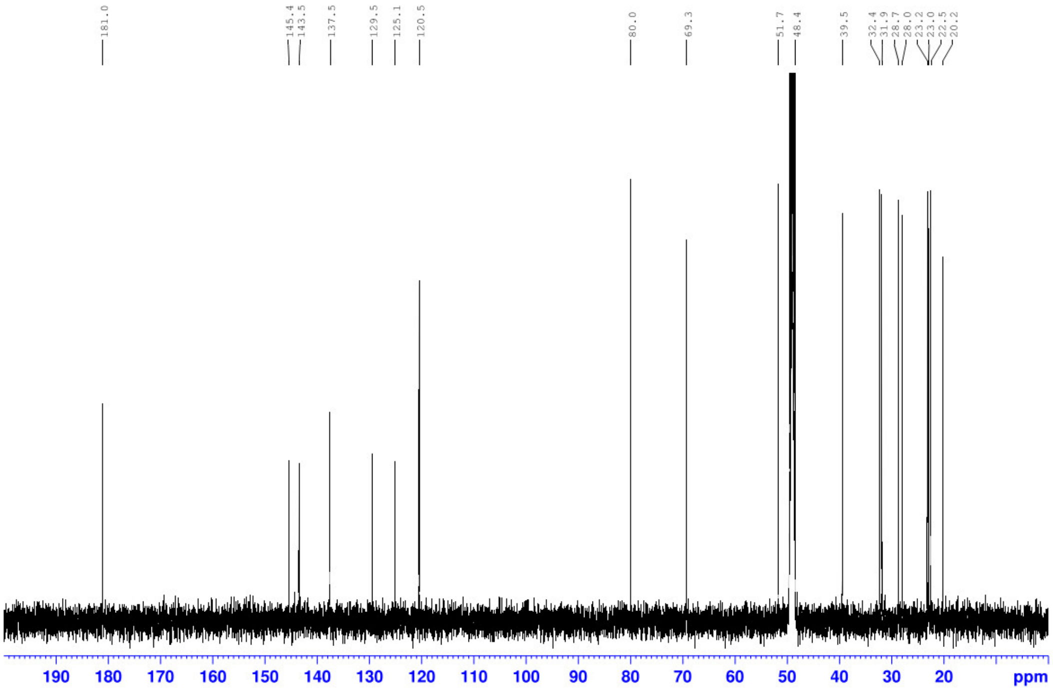


Figure S7. $^{13}$C-NMR spectrum of rosmanol (125 MHz in $CD_3OD$).

Compound **2**, obtained as white powder, had the molecular formula $C_{20}H_{26}O_4$ on basis of HR-ESI-MS *m/z* 329.1745 $[M-H]^-$. $^{1}$H-NMR (500 MHz, $CD_3OD$), $\delta_H$: 6.69 (1H, s, H-14), 5.42 (1H,

d, $J$ = 2.0 Hz, H-7), 3.22 (1H, m, H-15), 2.79 (1H, br d, $J$ = 14.5 Hz, H-1β), 2.54 (1H, td, $J$ = 14.0, 4.5 Hz, H-1α), 2.18 (1H, m, H-6α), 1.83 (2H, m, H-2β, 6β), 1.68 (1H, dd, $J$ = 11.0, 6.0 Hz, H-5), 1.61 (1H, m, H-3β), 1.51 (1H, m, H-2α), 1.31 (1H, td, $J$ = 13.5, 3.5 Hz, H-3α), 1.19 (3H, d, $J$ = 7.0 Hz, H-16), 1.18 (3H, d, $J$ = 7.0 Hz, H-17), 0.88 (3H, s, H-18), 0.87 (3H, s, H-19). $^{13}$C-NMR (125 MHz, $CD_3OD$), $\delta_C$: 30.1 (C-1), 20.0 (C-2), 42.2 (C-3), 35.5 (C-4), 47.0 (C-5), 30.9 (C-6), 79.7 (C-7), 133.3 (C-8), 123.0 (C-9), 49.8 (C-10), 144.7 (C-11), 144.2 (C-12), 136.1 (C-13), 112.5 (C-14), 28.0 (C-15), 23.2 (C-16), 20.1 (C-17), 32.2 (C-18), 23.2 (C-19), 179.3 (C-20). The NMR spectral data of **2** were in good agreement with those of carnosol reported in the reference, so was identified as carnosol.

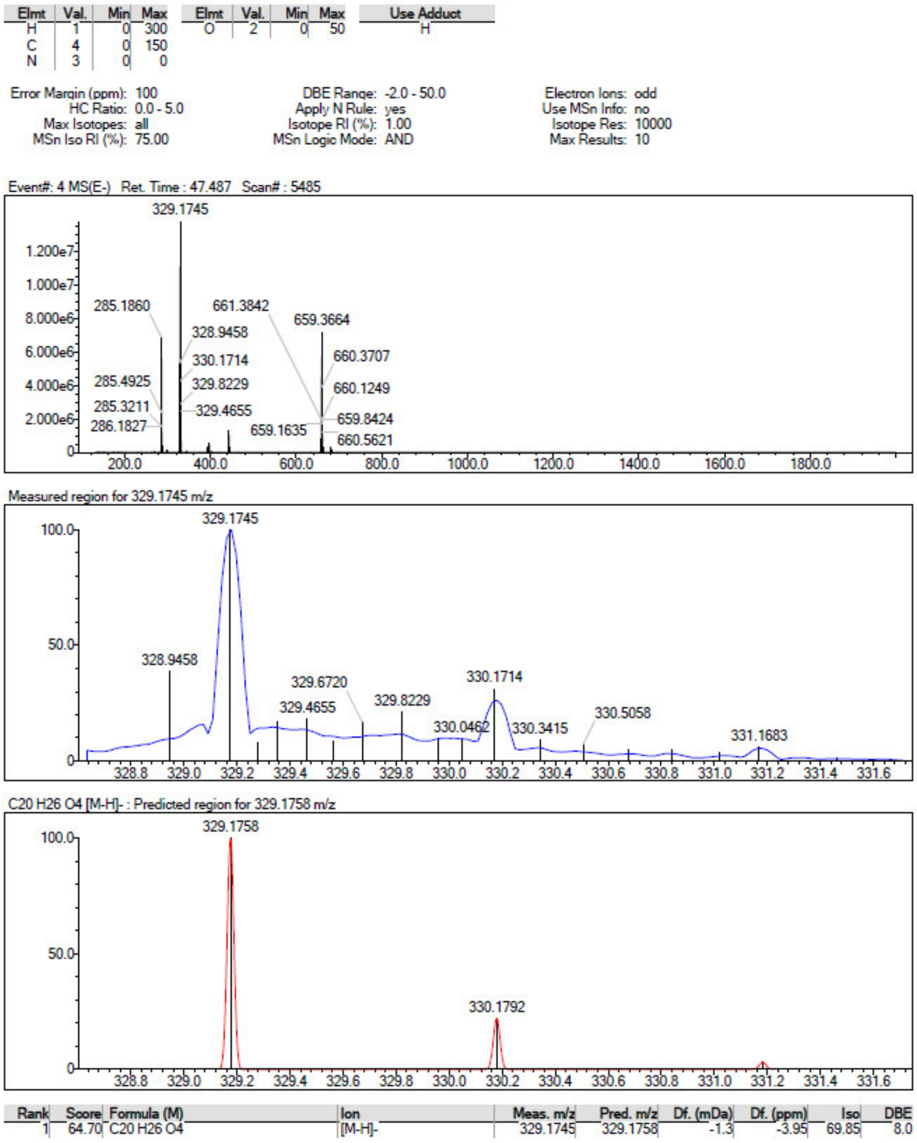


Figure S8. HR-ESI-MS spectrum of carnosol (**2**).

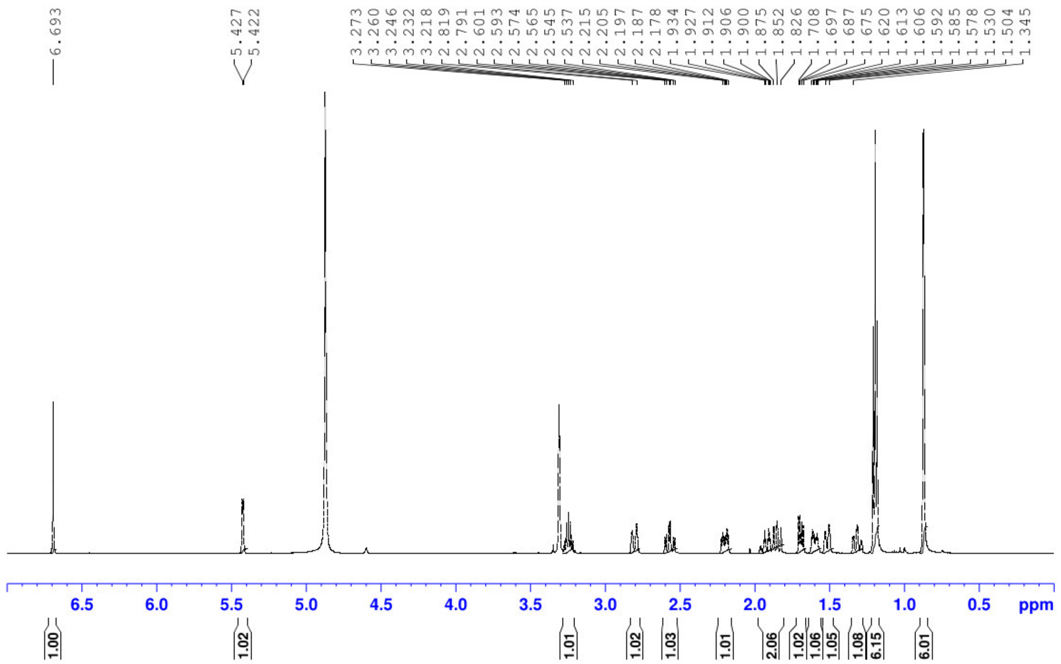


Figure S9. $^{1}$H-NMR spectrum of carnosol (**2**) (500 MHz in $CD_3OD$).

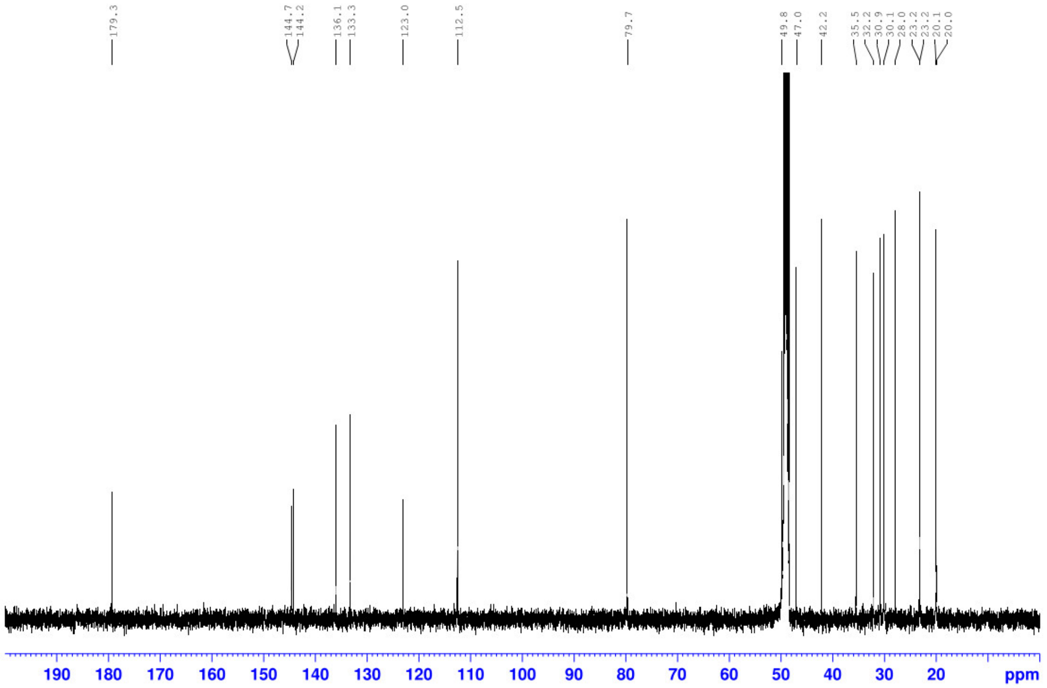


Figure S10. $^{13}$C-NMR spectrum of carnosol (**2**) (125 MHz in $CD_3OD$).

Table S1

Effects of rosmanol, carnosol and their combination on body weight in CIA DBA/1 mice

| Groups | Body weight (g, n=6) | | | | |
|---|---|---|---|---|---|
| | d0 | d21 | d28 | d35 | d42 |
| Normal | 18.9±0.7 | 21.7±0.8 | 22.5±1.0 | 23.4±1.2** | 24.1±1.3** |
| CIA model | 19.0±0.6 | 21.5±0.9 | 21.3±0.9 | 21.0±1.0 | 20.8±1.2 |
| R | 18.8±0.7 | 21.4±0.8 | 21.5±0.9 | 21.5±0.9 | 21.7±1.0 |
| C | 18.7±0.7 | 21.4±0.8 | 21.6±1.0 | 21.9±1.1 | 22.1±1.2 |
| R + C | 19.1±0.6 | 21.5±0.9 | 21.8±1.1 | 22.3±1.0* | 22.7±1.3* |

Compare to CIA model group,* $p<0.05$, **$p<0.01$.
